# Long-term PM2.5 exposure and new-onset lung diseases among middle-aged and older adults in China: a retrospective cohort study from CHARLS

**DOI:** 10.3389/fpubh.2025.1549830

**Published:** 2025-05-07

**Authors:** Xiaojun Wu, Kai Zhang, Daihua Yu, Jingjing Li

**Affiliations:** ^1^Department of General Surgery, Xi’an No.3 Hospital, The Affiliated Hospital of Northwest University, Xi’an, China; ^2^Department of Intensive Care Unit, Xi’an No.3 Hospital, The Affiliated Hospital of Northwest University, Xi’an, China

**Keywords:** long-term, PM2.5 exposure, new-onset lung diseases, middle-aged and older adults, China

## Abstract

**Background:**

Air pollution caused by ambient fine particulate matter (≤ 2.5 μm) adversely affects human health. Previous studies have shown that PM2.5 exposure increases the risk of lung diseases. However, the relationship between long-term PM2.5 exposure and new-onset lung diseases among middle-aged and older adults in China is still unclear.

**Methods:**

We conducted a retrospective cohort study through the China Health and Retirement Longitudinal Study (CHARLS) and Science Data Bank (ScienceDB). The logistic regression model and restricted cubic spline (RCS) were used to explore the relationship between long-term PM2.5 exposure and new-onset lung diseases. To further increase the robustness of the results, we performed sensitivity and subgroup analyses.

**Results:**

A total of 10,707 patients were included in this study. The 10,707 patients were divided into two groups: without new-onset lung diseases (*n* = 9,019) and with new-onset lung diseases (*n* = 1,688). The results of multivariate analysis showed that per 1 ug/m^3^ increase in annual PM2.5 concentration, the risk of new lung diseases increased by 0.3%. The results of the RCS showed that PM2.5 exposure increased the risk of new-onset lung diseases more significantly when the annual PM2.5 concentration was greater than 48.5 ug/m^3^. Sensitivity analysis and subgroup analysis also confirmed the reliability of the results.

**Conclusion:**

PM2.5 exposure increases the risk of new-onset lung diseases among middle-aged and old adults in China, especially when the concentration of PM2.5 > 48.5 ug/m^3^. Our study established an empirical foundation for refining PM2.5 emission regulations, developing age-stratified screening protocols for incident pulmonary diseases, and advancing mechanistic investigations into PM2.5-induced lung pathology.

## 1 Introduction

Air pollution caused by ambient fine particulate matter (PM2.5) has been widely recognized as a critical threat to global public health, with well-documented sources including industrial emissions, vehicle exhaust, and fossil fuel combustion (1–3). Epidemiological evidence consistently demonstrates that PM2.5 exposure elevates risks of various pulmonary pathologies. For instance, longitudinal data from Wang et al. (4) revealed an 11% increased emphysema risk per 2 μg/m^3^ PM2.5 increment over a decade, while Nakhjirgan et al.’s (5) cohort study identified a significant association between PM2.5 exposure and chronic obstructive pulmonary disease (COPD) development [HR = 1.032, 95% CI: 1.004–1.061] – a leading contributor to PM2.5-related mortality worldwide (5–7). Furthermore, emerging evidence links PM2.5 exposure to asthma exacerbation, lung carcinogenesis, and accelerated progression of idiopathic pulmonary fibrosis (8–10). Importantly, longitudinal declines in pulmonary function parameters such as forced vital capacity (FVC) and forced expiratory volume in 1 s (FEV₁) have been observed across all age groups exposed to chronic PM2.5 pollution (11, 12). Middle-aged and older adults represent a vulnerable population due to cumulative lifetime exposure and age-related physiological decline, including diminished pulmonary reserve and impaired pollutant clearance (13, 14). This susceptibility is particularly concerning in China, where severe PM2.5 pollution intersects with a substantial aging population (14, 15).

The China Health and Retirement Longitudinal Study (CHARLS) is a nationwide study that started in 2011. It includes data from more than 17,000 people nationwide and makes the data public after every 2–3 years of follow-up (16, 17). CHARLS has now been updated to 2020 (18). The Science Data Bank (ScienceDB), developed by the computer network information center of the Chinese Academy of Science, can provide scientific data-sharing services to researchers (19). This study aimed to explore the relationship between long-term PM2.5 exposure and new-onset lung disease among middle-aged and older adults in China through CHARLS and ScienceDB. This study investigated age-specific risks of new-onset lung diseases associated with varying levels of PM2.5 exposure, providing critical evidence to inform air quality management policies, offering references for developing age-specific screening protocols for emerging pulmonary conditions, and supporting empirical foundations for mechanistic investigations into PM2.5-related pulmonary pathogenesis.

## 2 Methods

### 2.1 Study design and inclusion of patients

**Figure 1:**
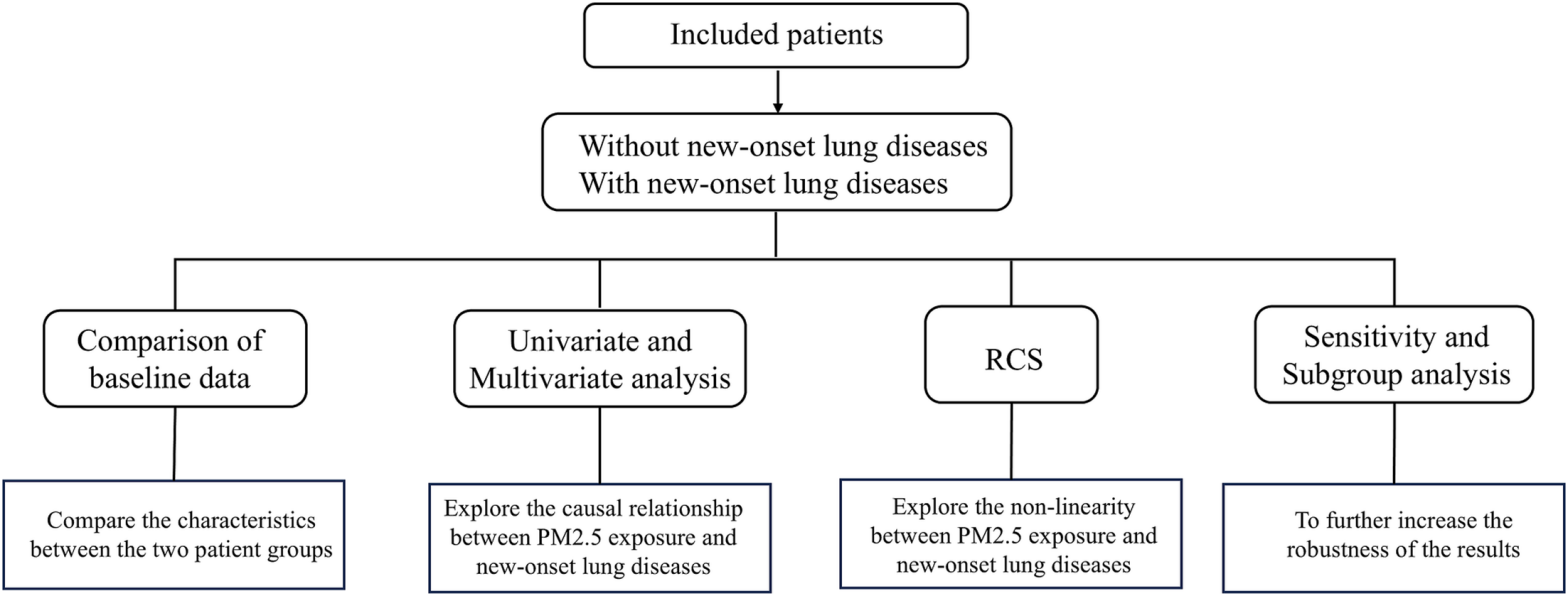
Schematic outline of this study.

We conducted this study using the CHARLS. Since most cities in China began the detection of PM2.5 in 2013, this study started with CHARLS 2013. This retrospective cohort study enrolled participants meeting strict inclusion/exclusion criteria, categorizing them into Patients with new-onset lung diseases and without new-onset lung diseases groups. Risk factor analysis for new-onset lung disease was performed after baseline data comparison (Figure 1).

Inclusion criteria of patients were as follows: (1) age ≥ 45 years, (2) patients without lung diseases in 2013, (3) patients with PM2.5 and follow-up data until 2020.

Exclusion criteria of patients were as follows: (1) age < 45 years, (2) patients with lung diseases in 2013, (3) patients without other related data (gender, insurance, etc.), (4) patients without follow-up data until 2020, and (5) patients without PM2.5 data.

### 2.2 Variable extraction

CHARLS provides several questionnaires from which we can obtain relevant data. The demographic background questionnaire included age, marital status, education level, and location data. The health status and functioning questionnaire provides data on gender, lung diseases (chronic bronchitis, emphysema, and pulmonary heart disease, but not lung cancer), chronic diseases (defined as one or more of hypertension, diabetes, coronary heart disease, asthma, arthritis, stroke, liver disease, kidney disease, etc.), smoking, and drinking. Insurance questionnaires provide data on insurance (Participation in insurance is defined as enrollment in at least one type of coverage, including but not limited to medical insurance, commercial insurance, or other analogous insurance schemes). The exit questionnaire provides data on follow-up information. New-onset lung diseases (chronic bronchitis, emphysema, and pulmonary heart disease, et.al, but not lung cancer) were defined as cases newly diagnosed during follow-up among participants without a prior history of lung disease. New-onset lung diseases and chronic diseases were diagnosed based on clinic visits and doctor-diagnosed conditions.

### 2.3 PM2.5 data

We obtained PM2.5 data from the Science Data Bank (ScienceDB).^1^ Liu et al. (20) collected global ground-based PM2.5 concentration data at Washington University. The annual average PM2.5 concentration data set of 342 administrative units in China can be obtained through ArcGIS software. The dataset was linearly correlated with the data published by the Chinese government, and the goodness of fit (R^2^) was above 0.8.

### 2.4 Statistical analysis

In this study, count data were described using numbers and percentages. A baseline comparison of counting data was performed using the chi-square test, and grade data was performed using the Mann–Whitney U test. The logistic regression model was used for univariate and multivariate analysis, with PM2.5 as the measurement data. Restricted cubic spline (RCS) was used to further explore the relationship between PM2.5 exposure and new-onset lung diseases. We performed sensitivity analysis through three models and subgroup analysis of age and chronic disease variables to further increase the robustness of the results. Statistical analyses were performed using SPSS 23.0 (SPSS Inc., Chicago, IL, USA) and R package (version 4.2.1).”rms” package was used to perform RCS in R Studio. *p* < 0.05 was considered statistically significant.

## 3 Results

### 3.1 Inclusion of patients

**Figure 2:**
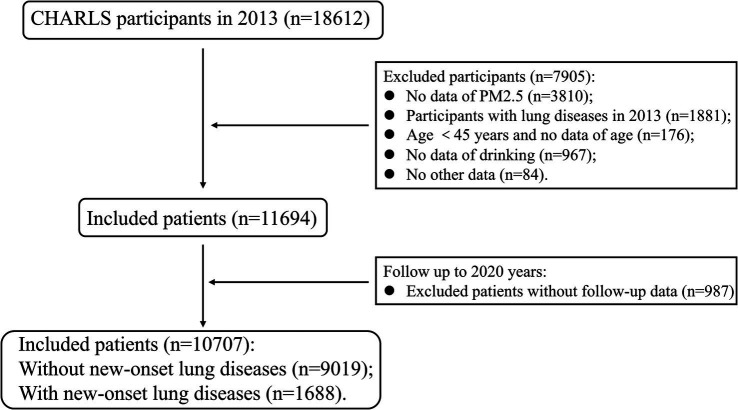
Flow chart of patient inclusion in this study.

CHARLS included a total of 18,612 individuals in 2013. We excluded 1,881 individuals who had lung disease in 2013. After combining the data from the ScienceDB through address information, 3,810 individuals without PM2.5 information were excluded. Then, we excluded 1,227 individuals without related information, and the remaining 11,654 patients were followed up. The endpoint of follow-up was the occurrence of new-onset lung diseases, and the follow-up period was from the time of inclusion to the occurrence of the endpoint event. After excluding 987 patients lost to follow-up, we finally included 10,707 patients in 2020. The 10,707 patients were divided into two groups based on whether the endpoint event occurred: without new-onset lung diseases (n = 9,019) and with new-onset lung diseases (*n* = 1,688) (Figure 2).

### 3.2 Comparison of baseline data

Patients with new-onset lung diseases had a higher proportion of age ≥ 65 (35.2% vs. 29.3%, *p* < 0.001), more male (51.3% vs. 45.7%, *p* < 0.001), more single (14.5% vs. 12.0%, *p* = 0.005), more illiteracy (49.9% vs. 45.9%, *p* < 0.001), more smokers (51.0% vs. 42.0%, *p* < 0.001), and more drinkers (49.8% vs. 44.4%, *p* < 0.001), more chronic diseases (80.7% vs. 67.2%, *p* < 0.001) (Table 1).

**Table 1:** Basic information in the baseline.

Characteristics	Total	Without lung diseases	With lung diseases	*p*
Number (%)	10,707 (100%)	9,019 (84.2%)	1,688 (15.8%)	
Age (*n*, %)				<0.001
<65	7,473 (69.8%)	6,380 (70.7%)	1,093 (64.8%)	
≥65	3,234 (30.2%)	2,639 (29.3%)	595 (35.2%)	
Gender (*n*, %)				<0.001
Male	4,984 (46.5%)	4,118 (45.7%)	866 (51.3%)	
Female	5,723 (53.5%)	4,901 (54.3%)	822 (48.7%)	
Marital status (*n*, %)				0.005
Single	1,325 (12.4%)	1,081 (12.0%)	244 (14.5%)	
Married	9,382 (87.6%)	7,938 (88.0%)	1,444 (85.5%)	
Residence (*n*, %)				0.052
Rural	6,933 (64.8%)	5,805 (64.4%)	1,128 (66.8%)	
Urban	3,774 (35.2%)	3,214 (35.6%)	560 (33.2%)	
Educational level (*n*, %)				<0.001
Illiteracy	4,981 (46.5%)	4,139 (45.9%)	842 (49.9%)	
Primary school	2,298 (21.5%)	1,906 (21.1%)	392 (23.2%)	
Middle school	2,244 (21.0%)	1,943 (21.5%)	301 (17.8%)	
High school and above	1,184 (11.1%)	1,031 (11.4%)	153 (9.1%)	
Smoking (yes, *n*, %)	4,662 (43.5%)	3,786 (42.0%)	876 (51.9%)	<0.001
Drinking (yes, *n*, %)	4,846 (45.3%)	4,006 (44.4%)	840 (49.8%)	<0.001
Insurance (yes, *n*, %)	10,483 (97.9%)	8,832 (97.9%)	1,651 (97.8%)	0.755
Chronic diseases (yes, *n*, %)	7,426 (69.4%)	6,063 (67.2%)	1,363 (80.7%)	<0.001

### 3.3 Univariate and multivariate analysis

**Figure 3:**
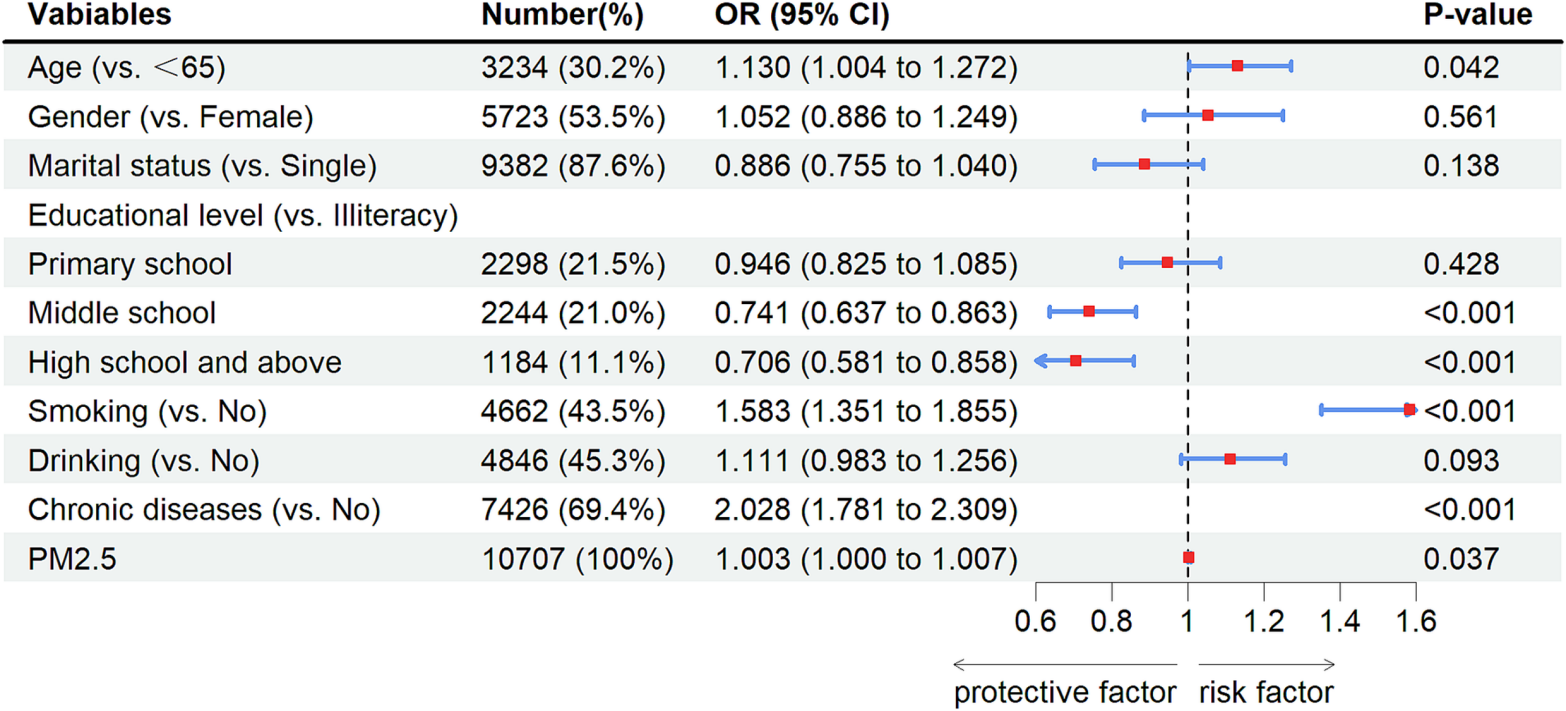
Forest plot of multivariate analysis. OR, odd ratio; CI, confidence interval.

The results of univariate analysis showed that there were statistical differences in age, gender, marital status, educational level, smoking, drinking, and chronic diseases (*p*-values lower than 0.05). However, there was no statistical difference in PM2.5 exposure (measurement data) (OR = 1.003, 95% CI: 0.999–1.006, *p* = 0.104). Even if it was not statistically significant, we conducted a multivariate analysis of the PM2.5 variables using measurement data (Table 2). The results of multivariate analysis showed that age ≥ 65 years (OR: 1.130, 95% CI: 1.004–1.2772, *p* = 0.042), smoking (OR: 1.583, 95% CI: 1.351–1.855, *p* < 0.001), chronic diseases (OR: 2.028, 95% CI: 1.781–2.309, *p* < 0.001), PM2.5 exposure (OR: 1.003, 95% CI: 1.000–1.007, *p* = 0.037) increased risks of new-onset lung diseases and more educational levels decreased risk of new-onset lung diseases: middle school (OR:0.741, 95%CI: 0.637–0.863, *p* < 0.001), high school and above (OR:0.706, 95%CI:0.581–0.858, *p* < 0.001) (Figure 3).

**Table 2:** Univariate and Multivariate logistic regression analysis.

Characteristics	Univariate		Multivariate	
	OR (95% CI)	*p*	OR (95% CI)	*p*
Age (vs. <65)	1.316 (1.179–1.469)	<0.001	1.130 (1.004–1.272)	0.042
Gender (vs. Female)	0.798 (0.719–0.885)	<0.001	1.052 (0.886–1.249)	0.561
Marital status (vs. Single)	0.806 (0.694–0.936)	0.005	0.886 (0.755–1.040)	0.138
Residence (vs. Rural)	0.897 (0.803–1.001)	0.052		
Educational level (vs. Illiteracy)				
Primary school	1.011 (0.886–1.153)	0.871	0.946 (0.825–1.085)	0.428
Middle school	0.762 (0.661–0.878)	<0.001	0.741 (0.637–0.863)	<0.001
High school and above	0.729 (0.606–0.878)	0.001	0.706 (0.581–0.858)	<0.001
Smoking (vs. No)	1.491 (1.344–1.655)	<0.001	1.583 (1.351–1.855)	<0.001
Drinking (vs. No)	1.240 (1.117–1.376)	<0.001	1.111 (0.983–1.256)	0.093
Insurance (vs. No)	0.945 (0.661–1.350)	0.755		
Chronic diseases (vs. No)	2.045 (1.798–2.326)	<0.001	2.028 (1.781–2.309)	<0.001
PM2.5	1.003 (0.999–1.006)	0.104	1.003 (1.000–1.007)	0.037

OR, odd ratio; CI, confidence interval.

### 3.4 Results of RCS

**Figure 4:**
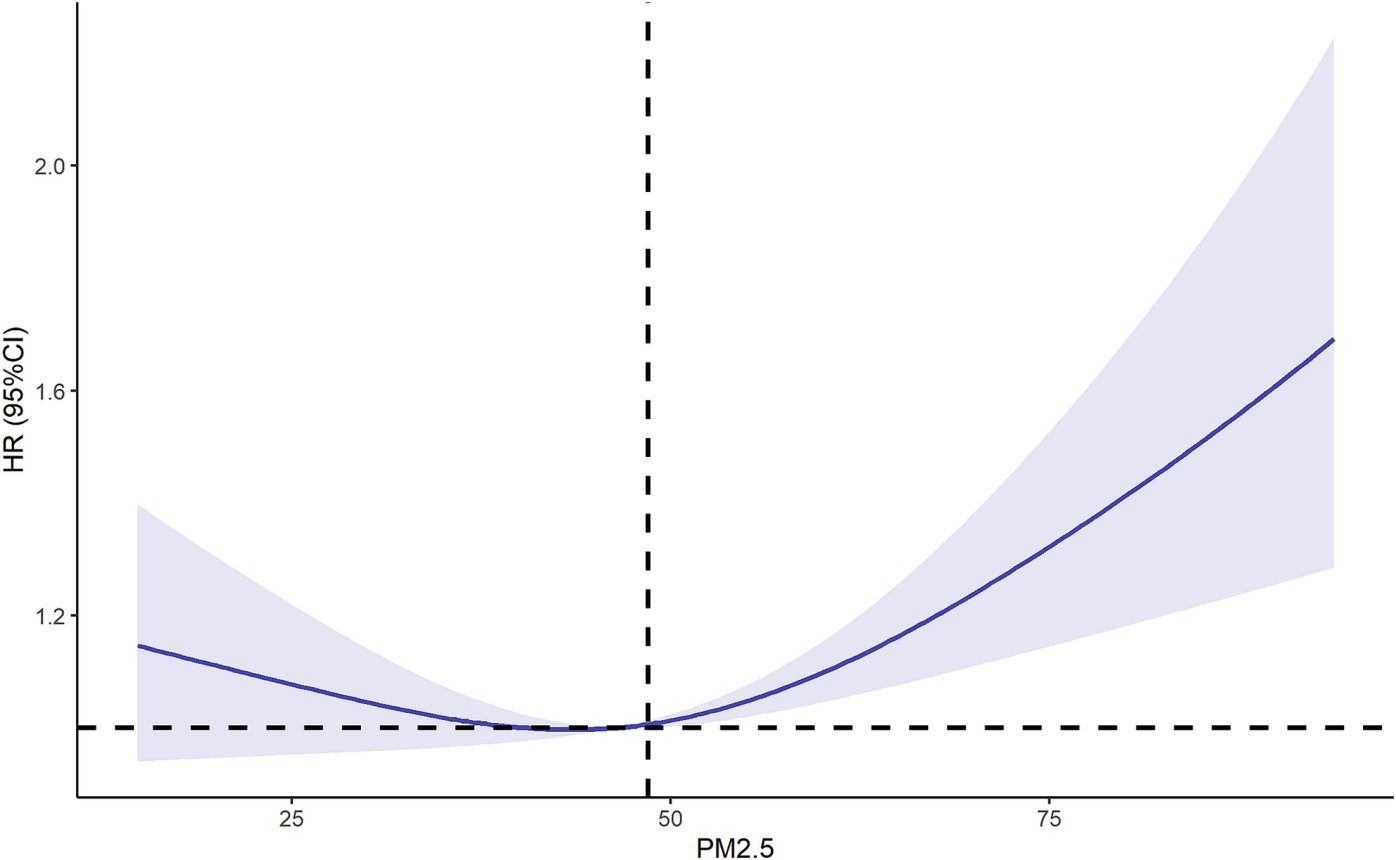
Restricted cubic spline of relationship between PM2.5 and new-onset lung diseases. Adjusting by age, gender, marital status, residence, educational level, smoking, drinking, insurance and chronic diseases. OR, odd ratio; CI, confidence interval.

RCS balance flexibility and stability by constraining tail linearity, avoid overfitting through low-degree polynomials, enable intuitive visualization, and support statistical inference, enhancing clinical interpretability. Then we used the RCS model to further explore the relationship between PM2.5 exposure and new-onset lung diseases. After adjusting for multiple variables, the results of RCS showed a positive association between PM2.5 exposure and new-onset lung diseases when the concentration of PM2.5 > 48.5 ug/m^3^ (P of non-linearity = 0.004) (Figure 4).

### 3.5 Results of the sensitivity analysis

We explored the relationship between PM2.5 exposure and new-onset lung diseases through multiple models in the form of measurement data and count data (Q1: PM2.5 < 48.5 ug/m^3^; Q2: PM2.5 ≥ 48.5 ug/m^3^), respectively to further increase the robustness of the results (Table 3). Model 1: adjusted by age, gender, marital status, residence, educational level; Model 2: additional adjusted by smoking and drinking; Model 3: additional adjusted by insurance and chronic diseases.

**Table 3:** Association between PM2.5 and new-onset lung diseases in different model.

Characteristics	Model 1		Model 2		Model 3	
	OR (95%CI)	*p*	OR (95%CI)	*p*	OR (95%CI)	*p*
PM2.5	1.004 (1.000–1.007)	0.031	1.003 (1.000–1.007)	0.038	1.003 (1.000–1.007)	0.037
PM2.5 (vs.Q1)	1.232 (1.109–1.369)	<0.001	1.228 (1.104–1.364)	<0.001	1.227 (1.103–1.365)	<0.001

OR, odd ratio; CI, confidence interval. Model 1: adjusted by age, gender, marital status, residence, educational level; Model 2: additional adjusted by smoking and drinking; Model 3: additional adjusted by insurance and chronic diseases (Q1: PM2.5 < 48.5ug/m^3^; Q2:PM2.5 ≥ 48.5ug/m^3^).

All models showed that PM2.5 exposure increased the risk of new-onset lung diseases when PM2.5 was used as measurement data. Model 1 (OR: 1.004, 95% CI: 1.000–1.007, *p* = 0.031). Model 2 (OR: 1.003, 95% CI: 1.000–1.007, *p* = 0.038). Model 3 (OR: 1.003, 95% CI: 1.000–1.007, *p* = 0.037).

All models also showed that PM2.5 exposure increased the risk of new-onset lung diseases when PM2.5 was used as count data (Q1 as reference). Model 1 (OR: 1.232, 95% CI: 1.109–1.369, *p* < 0.001). Model 2 (OR: 1.228, 95% CI: 1.104–1.364, *p* < 0.001). Model 3 (OR: 1.227, 95% CI: 1.103–1.365, *p* < 0.001).

### 3.6 Results of the subgroup analysis

We also used three models for subgroup analysis in age and smoking variables, with PM2.5 as the measurement and count data (Q1: PM2.5 < 48.5 ug/m^3^; Q2: PM2.5 ≥ 48.5 ug/m^3^), respectively (Table 4). The P results for interaction showed no interaction (*p* > 0.05).

**Table 4:** Subgroup analysis of the association between PM2.5 and new-onset lung diseases by different models.

Characteristics	Model 1		Model 2		Model 3		
	OR (95%CI)	*p*	OR (95%CI)	*p*	OR (95%CI)	*p*	P for interaction
PM2.5 (Measurement)							
Age							0.090
<65	1.002 (0.998–1.006)	0.382	1.002 (0.998–1.006)	0.382	1.002 (0.998–1.006)	0.302	
≥65	1.007 (1.002–1.013)	0.012	1.007 (1.001–1.013)	0.017	1.007 (1.001–1.012)	0.021	
Smoking							0.071
No	1.000 (0.995–1.005)	0.986	1.000 (0.996–1.005)	0.897	1.000 (0.996–1.005)	0.874	
Yes	1.006 (1.002–1.011)	0.006	1.006 (1.002–1.011)	0.006	1.006 (1.002–1.011)	0.005	
PM2.5 (vs.Q1)							
Age							0.222
<65	1.180 (1.036–1.344)	0.013	1.186 (1.040–1.351)	0.011	1.198 (1.050–1.366)	0.007	
≥65	1.342 (1.120–1.607)	0.001	1.322 (1.103–1.585)	0.003	1.310 (1.092–1.571)	0.004	
Smoking							0.451
No	1.173 (1.010–1.363)	0.037	1.190 (1.023–1.383)	0.024	1.188 (1.020–1.382)	0.026	
Yes	1.279 (1.101–1.485)	0.001	1.278 (1.101–1.484)	0.001	1.283 (1.104–1.491)	0.001	

OR, odd ratio; CI, confidence interval. Model 1: adjusted by age, gender, marital status, residence, educational level; Model 2: additional adjusted by smoking and drinking; Model 3: additional adjusted by insurance and chronic diseases (Q1: PM2.5 < 48.5ug/m^3^; Q2:PM2.5 ≥ 48.5ug/m^3^).

PM2.5 as measurement data: when age ≥ 65 years, PM2.5 exposure increases the risks of new-onset lung diseases (*p* < 0.05). In subgroups without smoking, PM2.5 exposure increased the risks of new-onset lung diseases (*p* < 0.05).

PM2.5 as count data: PM2.5 exposure increased the risks of new-onset lung diseases (Q1 as a reference) in both subgroups of age < 65 years (*p* < 0.05) and age ≥ 65 years (*p* < 0.05). In subgroups with and without smoking, PM2.5 exposure increased the risks of new-onset lung diseases (Q1 as a reference) (*p* < 0.05).

## 4 Discussion

PM2.5 exposure increases the risks of chronic lung diseases, which has been widely reported in high-income countries (20–22). However, PM2.5 detection in China starts late, and the relationship between PM2.5 exposure and new-onset lung diseases among middle-aged and older adults remains unclear.

Our findings demonstrated a significant association between long-term PM2.5 exposure and the incidence of new-onset lung diseases among middle-aged and older adults. The results of univariate analysis showed PM2.5 exposure did not increase the risk of new-onset lung diseases (*p* = 0.104). The results of multivariate analysis showed that per 1 ug/m^3^ increase in annual PM2.5 concentration, the risk of new lung diseases increased by 0.3%(*p* = 0.037). This phenomenon may occur because the effect of PM2.5 was masked by age. As age increased, cumulative exposure to PM2.5 became longer. When age and PM2.5 were included together in a multivariate analysis, the OR for age decreased, and the effect of PM2.5 was no longer obscured by age. The results of the RCS showed that PM2.5 exposure increased the risk of new-onset lung diseases more significantly when the annual PM2.5 concentration was greater than 48.5 ug/m^3^. Sensitivity analysis and subgroup analysis also confirmed the reliability of the results.

Our results were consistent with previous research (23, 24): PM2.5 exposure elevated the risk of respiratory diseases such as asthma. Our study delved deeper into the dose–response relationship between PM2.5 exposure levels and pulmonary outcomes, proposing a threshold of 48.5 μg/m^3^ as a critical upper limit for targeted air quality regulation. While current annual PM2.5 levels in high-income countries generally remain below 48.5 μg/m^3^, our findings hold particular relevance for informing region-specific policy formulation in low-and middle-income countries (LMICs). China’s national standards (annual: 35 μg/m^3^, 24-h: 75 μg/m^3^) reflect substantial progress in air quality governance (13, 25). However, persistent disparities in regional development within China necessitate flexibility. This study provides evidence-based phased implementation strategies for provinces facing challenges in achieving national targets, supporting the implementation of regionally adaptable interim standards during transitional phases.

Some mechanism studies may explain the results: According to Fan et al. (26), PM2.5 exposure enhanced mitophagy via triphosphopyridine nucleotide oxidase 4 (NOX4)/ transcription factor nuclear factor erythroid 2-related factor 2 (Nrf2) redox imbalance, further increasing susceptibility to acute exacerbation of COPD (AECOPD). Research by Guo et al. (27) showed PM2.5 exposure up-regulated the expression of methyltransferase-like protein 16 (METTL16), subsequently altered N6-methyladenosine (m6A) modification, and finally microvascular injury in COPD. According to Zhang et al. (28), PM2.5 exposure induced lung injury in Wistar rats via activating AMP-activated protein kinase (AMPK)—sirtuin 1 (SIRT1)—peroxisome proliferator-activated receptor-*γ* coactivator 1α (PGC-1α) pathway. In conclusion, PM2.5 plays an important role in accelerating the progression of lung diseases by activating reactive oxygen species (ROS) and inflammation (29–31). Therefore, previous studies have provided new ideas for treating lung diseases through the anti-inflammatory and antioxidant effects of Resveratrol, Biochanin A Polyphenols, etc. (32–34).

This study advanced environmental epidemiology by establishing a novel age-stratified exposure-response framework. Particularly, the identification of differential susceptibility patterns across age deciles provided granular evidence for refining air quality standards, (b) necessitated age-optimized early detection strategies in clinical practice, and (c) pinpointed critical windows for molecular epidemiological research on PM2.5-induced pulmonary damage.

There were some drawbacks to this study. First, this study was retrospective, and there were inevitable biases in the data collection process. Second, we explored only the effect of outdoor PM2.5 exposure on new-onset lung diseases. However, the impact of indoor PM2.5 could not be investigated because the data could not be obtained. The relationship between indoor PM2.5 exposure and new-onset lung diseases warrants further investigation in future studies. Finally, the PM2.5 data used in this study may not fully reflect the true PM2.5 exposure of an individual, as the location of an individual may vary. Nevertheless, our findings provided critical evidence-based insights to inform the formulation of regionally adaptable PM2.5 emission standards.

## 5 Conclusion

Our study identifies a critical PM2.5 threshold of 48.5 μg/m^3^ for incident pulmonary diseases in Chinese middle-aged and older adults. This threshold aligns with regional pollution disparities and aging-specific vulnerabilities, suggesting its utility as a transitional target for provinces struggling to meet China’s current national standard (35 μg/m^3^). Implementing regionally adaptive policies. Mechanistic research associated with PM2.5 exposure and new-onset lung diseases also should be performed to guide clinical prevention and treatment.

## Data Availability

The original contributions presented in the study are included in the article/supplementary material, further inquiries can be directed to the corresponding author.

## Footnotes

^1^ https://www.scidb.cn/en
